# The Transactions of the International Hahnemannian Association

**Published:** 1891-03

**Authors:** J. W. Thomson

**Affiliations:** 114 W. 16th Street, New York


### THE TRANSACTIONS OF THE INTERNATIONAL HAHNEMANNIAN ASSOCIATION.

To the Editors of The Homœopathic Physician.

Gentlemen:—In the August number of The Homœopathic Physician for 1890 appeared a brief report of the discussion which followed Dr. W. L. Reed's paper on Albuminuria. In the proceedings of the I. H. A. which came to hand February 19th, Dr. Wm. A. Hawley's name, who spoke twice, is not even mentioned. There is interpolated a speech by Dr. H. C. Allen after my remarks. Had such remarks been made they would not have been allowed to pass unchallenged. The error also occurs on page 50 of the January number of the *Advance.*

In the discussion on my paper, which is called, "A Clinical Case," instead of being known by the rubric I gave, there were remarks both by Dr. Bell and myself which are omitted.

A letter addressed to the New York *Sun*, which I read before the Association is also left out. And the whole of the discussion with reference to the publication of the transactions does not appear. J. W. THOMSON.

114 W. 16TH STREET, NEW YORK.

February 21st, 1891.

